# Age and Origin of the Founder Antithrombin Budapest 3 (p.Leu131Phe) Mutation; Its High Prevalence in the Roma Population and Its Association With Cardiovascular Diseases

**DOI:** 10.3389/fcvm.2020.617711

**Published:** 2021-02-05

**Authors:** Zsuzsanna Bereczky, Réka Gindele, Szilvia Fiatal, Marianna Speker, Tünde Miklós, László Balogh, Zoltán Mezei, Zsuzsanna Szabó, Róza Ádány

**Affiliations:** ^1^Division of Clinical Laboratory Science, Department of Laboratory Medicine, Faculty of Medicine, University of Debrecen, Debrecen, Hungary; ^2^Department of Public Health and Epidemiology, Faculty of Medicine, University of Debrecen, Debrecen, Hungary; ^3^Department of Cardiology and Cardiovascular Surgery, Faculty of Medicine, University of Debrecen, Debrecen, Hungary; ^4^Department of Laboratory Medicine, Faculty of Medicine, University of Debrecen, Debrecen, Hungary; ^5^Magyar Tudományos Akadémia - Debrecen Public Health Research Group, University of Debrecen, Debrecen, Hungary

**Keywords:** antithrombin deficiency, antithrombin Budapest 3, founder effect, Roma population, cardiovascular disease, thrombosis

## Abstract

**Background:** Antithrombin (AT) is one of the most important regulator of hemostasis. AT Budapest 3 (ATBp3) is a prevalent type II heparin-binding site (IIHBS) deficiency due to founder effect. Thrombosis is a complex disease including arterial (ATE) and venous thrombotic events (VTE) and the Roma population, the largest ethnic minority in Europe has increased susceptibility to these diseases partly due to their unfavorable genetic load. We aimed to calculate the age and origin of ATBp3 and to explore whether the frequency of it is higher in the Roma population as compared with the general population from the corresponding geographical area. We investigated the association of ATBp3 with thrombotic events in well-defined patients' populations in order to refine the recommendation when testing for ATBp3 is useful.

**Methods and Results:** Prevalence of ATBp3, investigated in large samples (*n* = 1,000 and 1,185 for general Hungarian and Roma populations, respectively) was considerably high, almost 3%, among Roma and the founder effect was confirmed in their samples, while it was absent in the Hungarian general population. Age of ATBp3—as calculated by analysis of 8 short tandem repeat sequences surrounding *SERPINC1*—was dated back to XVII Century, when Roma migration in Central and Eastern Europe occurred. In our IIHBS cohort (*n* = 230), VTE was registered in almost all ATBp3 homozygotes (93%) and in 44% of heterozygotes. ATE occurred with lower frequency in ATBp3 (around 6%); it was rather associated with AT Basel (44%). All patients with ATE were young at the time of diagnosis. Upon investigating consecutive young (<40 years) patients with ATE (*n* = 92) and VTE (*n* = 110), the presence of ATBp3 was remarkable.

**Conclusions:** ATBp3, a 400-year-old founder mutation is prevalent in Roma population and its Roma origin can reasonably be assumed. By the demonstration of the presence of ATBp3 in ATE patients, we draw the attention to consider type IIHBS AT deficiency in the background of not only VTE but also ATE, especially in selected populations as young patients without advanced atherosclerosis. We recommend including the investigation of ATBp3 as part of thrombosis risk assessment and stratification in Roma individuals.

## Introduction

Thrombosis is a common complex disease including arterial thrombotic diseases as myocardial infarction (MI), ischemic stroke (IS), or peripheral arterial occlusive disease and venous thrombotic events (VTE), which are the major contributors of morbidity and mortality in all developed countries (1). Antithrombin (AT) is a single-chain glycoprotein belonging to the SERPIN family, and it is one of the most important regulators of hemostasis (2). The mature protein, which is composed of 432 amino acids, has two glycoforms in the circulation, the major α- (90–95%) and a β-glycoform (<10%), the latter of which lacks *N*-glycosylation on Asn135 (Asn167 according to HGVS nomenclature, http://www.HGVS.org/varnomen) (3). Major targets of AT are serine-protease coagulation factors, and its effect is the most pronounced to thrombin and activated FX (FXa). Heparin, low molecular weight heparin, pentasaccharides, and heparan sulfate proteoglycan molecules are able to increase its inhibitory effect by more than 500-fold (4, 5). Heparin-binding site (HBS) of AT encoded by exon 2 of *SERPINC1* (the gene encoding AT) is responsible for the interaction between heparins and AT (2). Mutations in this region lead to functional AT deficiency, namely, type IIHBS with decreased heparin-AT interaction. Most frequent type IIHBS mutations are AT Basel (p.Pro73Leu), AT Padua (p.Arg79His), and AT Budapest 3 (p.Leu131Phe) (6–8). Heterozygous AT-deficient patients have a high thrombotic risk in general (9). The different AT deficiency subtypes, however, do not have the same clinical phenotype, and based on the results of some clinical studies, type IIHBS deficiency seems to exert a lower thrombotic risk (10–12). Additionally, the situation is even more complicated, since type IIHBS homozygotes present the most severe thrombotic symptoms among all AT-deficient patients (13). The first-line laboratory assay in the diagnosis of AT deficiency is a functional amidolytic test in which the inhibitory effect of AT on active FXa or thrombin is investigated in the presence of heparin (14, 15). This assay shows reduced AT activity in all AT deficiency types, including type II variants. Since progressive AT activity (performed by functional assay in the absence of heparin) is normal only in type IIHBS deficiency, this second-line test serves as an excellent tool for differentiating these patients from other AT-deficient individuals (16).

While thrombophilia testing, including AT deficiency, has its role in VTE, testing for thrombophilia in arterial thrombotic events (ATE) is rather controversial (17–20). Most of the guidelines are against inherited thrombophilia testing in ATE (21, 22). In unselected population, this approach has rationale. However, in a recent study, examining patients with MI with non-obstructive coronary arteries (MINOCA) thrombophilia was found in almost one-fourth of patients (23). According to a more recent concept of atherothrombosis, ATE and VTE have more in common than it was supposed earlier (24, 25). Based on the presence of the same structural elements of *ex vivo* arterial and venous thrombi and based on the findings of clinical studies in which dual pathway inhibition was beneficial, it is reasonable to suppose that certain risk factors of VTE may play a role also in arterial diseases (26–29).

Population-specific differences may also contribute to modify the general view of thrombophilia investigation in selected samples. Young individuals with ATE have different risk factor profile upon comparison with older patients in several clinical studies (30). The atherosclerotic burden is less remarkable in this group and not only the risk profile but also the clinical presentation may be different from older people (31). It seems that smoking has a more pronounced impact in the development of MI in the young, and among its several adverse effects, hypercoagulability seems to be the most important (32, 33). The presence of certain genetic determinants can also influence clinical decision making in certain populations. As an example, the prevalence of Factor V Leiden mutation (FVL, rs6025) shows high variability worldwide, and it is frequent in certain populations, while it is missing from others (34, 35). Beside AT Cambridge II, AT Basel and AT Budapest 3 (ATBp3) are the most frequent and well-known founder mutations in AT deficiency, which may increase the otherwise low prevalence of AT deficiency in the affected populations (6, 8). The founder effect of ATBp3 in the Hungarian population was confirmed previously by haplotype analysis, by investigating rs3138521, rs5877, rs5878, rs2227596, rs941989, rs1799876, rs677, and rs2227612 and short tandem repeat (STR) markers, namely *SERPINC1*-Alu5 and Alu8, D1S196, and D1S218 (8). In all ATBp3 individuals, the pathogenic “T” allele was associated with the same haplotype, while the normal “C” allele was associated with different haplotypes both in ATBp3 heterozygotes and in control subjects. ATBp3 homozygous patients shared one distinct Alu5 and Alu8 repeat number variations (ATT)_6_ and (ATT)_15_, respectively. The STR marker closer to *SERPINC1* (D1S218) showed bi-allelic distribution in ATBp3 homozygotes carrying (AC)_24_ and (AC)_25_ and D1S196 was variable. The age and origin of this mutation however have not been investigated, as yet.

The Roma ethnic group as the largest and most widespread minority group in Europe is one of the major subjects of ethnicity-based studies (36). The Roma population has significantly worse cardiovascular health as compared with the general population in the same country, where they settled down (37). Roma people have a higher cardiovascular morbidity rate, they may be younger at the time of first thrombotic episodes and their risk factor profile is much worse as compared with the majority population (38–40). Among modifiable risk factors, smoking, dyslipidemia, and obesity were found significantly more frequent in Roma people (40, 41). According to a number of papers, the genetic load of Roma population to atherosclerosis and thrombosis were significantly higher comparing it with the general reference population, and among thrombophilia factors, the FVL allele was more frequent (i.e., 11.12% in the Roma vs. 4.29% in the general population) in most recent studies (42, 43). Several other susceptibility allele frequencies were also significantly higher in the Roma population among SNPs investigated in the same cross-sectional study (43). These results suggest that the combination of high genetic susceptibility for thrombotic diseases with the presence of an unfavorable environmental risk factor profile makes this ethnic population extremely sensitive for these diseases. It was, however not investigated, if the founder ATBp3 mutation, which—as opposed to the previously mentioned SNPs each with small effect size—represents a severe thrombotic risk, may be frequent in the Roma population.

The aims of the present study were to explore the frequency of the founder ATBp3 mutation in the Roma general population and compared data with the general reference sample from the corresponding geographical area. We aimed to calculate the age and origin of the founder ATBp3 in the general population and to test our hypothesis of its Roma origin. We aimed to investigate the association of type IIHBS mutations, including the prevalent ATBp3, with VTE and ATE in well-defined populations to see whether it is relevant to give a recommendation for more extensive testing for AT deficiency in certain populations.

## Patients and Methods

### Study Population

#### Reference Groups for Genetic Epidemiology Studies

A large number of individuals (*n* = 1,000, median age 55, range 29–102 years, male/female 46.6/53.4%) representing the general Hungarian population were recruited in the framework of the Hungarian General Practitioners' Morbidity Sentinel Stations Program (44). Another group (*n* = 1,185, median age 40 years, range 18–87, male/female 41.0/59.0%) representing the Roma general population living in segregated colonies in Northeast Hungary was also recruited. The details of the sampling methodology and data collected are described elsewhere (41, 45). These two samples were considered as reference samples.

For confirmatory analysis in a separate study (46), healthy Hungarian individuals (*n* = 450, median age 34 years, range 18–68, male/female 38.6/61.4%) free from any cardiovascular disease and considered a healthy reference group were recruited. Except for moderate hypertension (HT) in their case histories (blood pressure between 145/90 and 165/95 mmHg), all chronic diseases were considered exclusion criteria. Roma individuals (*n* = 402, median age 44 years, range 18–77, male/female 26.6/73.4%) from the corresponding geographical area as the general Roma population were also recruited.

#### Patients' Groups Recruited for the Investigation of the Association Between Type IIHBS AT Deficiency and Thrombotic Diseases

To investigate the association of AT IIHBS deficiency and thrombotic diseases, different patients' groups were collected. First, *n* = 243 non-related genetically confirmed type IIHBS AT-deficient patients (index patients) diagnosed at our center between January 2007 and August 2020 and their affected family members (total *n* = 328), were involved. Inclusion criteria were low AT levels measured by anti-FXa heparin cofactor AT activity assay (hc-anti-FXa) and the confirmed type IIHBS mutation in the genetic test.

A second group including young adults with ST-elevation MI in their case histories below the age of 40 years was collected (*n* = 119, median age 36 years, male/female 79/21%) in order to investigate the association of hemostasis alterations with MI in this young age group, where the prevalence of occlusive arterial diseases was low. The diagnosis of MI based on the current guideline for universal definition of MI based on the biomarker detection plus the presence of clinical symptoms or ECG changes characteristics for myocardial ischemia or identification of coronary thrombus (47). Age-matched clinical control (CC) individuals (*n* = 101, median age 36 years, male/female 59/41%) were also recruited, who had undergone coronary angiography, but no coronary artery disease has been revealed and no MI was recorded in their case histories. Indication of coronary angiography for them was the clinical suspicion for stable angina, as they had at least one positive non-invasive test for assessment of myocardial ischemia. Detailed definitions of MI and CC patients' inclusion and diagnostic criteria and the circumstances of enrolling are described elsewhere (33).

Finally, a group consisting of consecutive, non-related patients with VTE in their case histories below the age of 40 was investigated (*n* = 110, median age 31 years, male/female 52/48%). Thrombosis was diagnosed and categorized into spontaneous and provoked according to guidance of the International Society of Thrombosis and Haemostasis (48). Clinical and laboratory data were collected as previously described (13). Patient clinical, laboratory, and genetic data were recorded in a database for further evaluation. As data collection by ethnic status is not allowed in health care services, we were not able to collect information about the ethnicity of the patients.

#### Ethical Approval

All enrolled individuals were informed about the study according to the study protocol and gave written informed consent. Ethical approval for the study was obtained from Committee of the Hungarian Scientific Council on Health (3166/2012/HER and NKFP/1/0003/2005; 8907-O/2011-EKU, 61327-2017/EKU). The study was approved by the Ethical Committee of University of Debrecen, Hungary (reference No. 2462-2006), and all performed procedures were in accordance with the 1964 Helsinki declaration and its later amendments.

### Laboratory Methods

Fasting blood samples were collected into 0.109 mol/L citrate vacutainer tubes (Beckton Dickinson, Franklin Lakes, NJ, USA) at least 3 months after the acute thrombotic episode or coronarography (if relevant) and stored at −80°C until use. Native blood samples were also collected and stored at −80°C. For diagnosing AT deficiency, hc-anti-FXa and progressive (p-anti-FXa) AT activity were measured (Labexpert Antithrombin H+P, Labexpert Ltd, Debrecen, Hungary, reference intervals 80–120 and 82–118%, respectively) on a Siemens BCS-XP coagulometer. AT antigen was measured by immunonephelometry (Siemens, N Antiserum to Human Antithrombin III, Marburg, Germany; reference interval 0.19–0.31 g/L). Protein C activity and free protein S antigen were measured by commercially available assays from Siemens (PC chromogenic and Innovance free PS antigen). Fibrinogen was measured by the Clauss method using Labexpert reagent. Measurement of other plasma or serum parameters was executed by routine methods using Roche reagents and instruments (Roche Diagnostics GmbH, Mannheim, Germany). DNA was isolated from the buffy coat of citrated blood samples by QIAamp DNA Blood Mini Kit (Qiagen, Hilden, Germany) in case of patients' samples. DNA was isolated using the MagNA Pure LC system (Roche Diagnostics) with a MagNA Pure LC DNA Isolation Kit-Large Volume according to the manufacturer's instructions in case of the reference samples.

Sanger sequencing searching for *SERPINC1* mutations in the exons, the flanking intronic regions and in the promoter was executed by ABI3130 Genetic Analyzer and Sequencing Analysis 5.4 software (Thermo Fisher Scientific, Carlsbad, CA, USA) in AT-deficient group and in case of MI, CC, and VTE samples upon abnormal hc-anti-FXa AT activity results according to protocols described earlier (8). ATBp3 mutation, FVL, and the prothrombin 20210G>A polymorphisms were determined according to protocols developed in our laboratory on Roche LightCycler 480 instrument by using real-time PCR and melting curve analysis in all groups.

**Figure 1:**
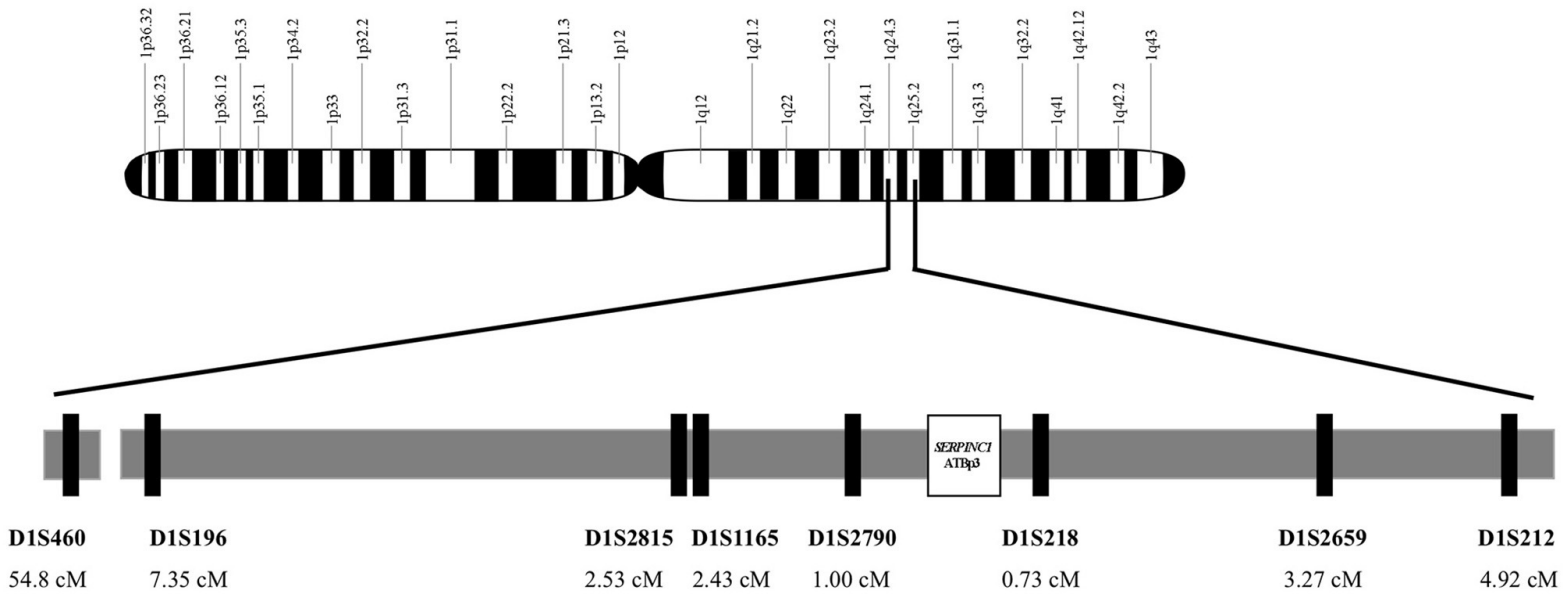
Genomic map of chromosome 1. Localization and position of *SERPINC1* gene and the analyzed short tandem repeat markers on region 1q24-25. Short tandem repeat markers are D1S460 (CA)_*n*_, D1S196 (AC)_*n*_, D1S2815 (CA)_*n*_, D1S1165(CTTT)_*n*_, and D1S2790 (CA)_*n*_ locate proximal to *SERPINC1* and D1S218 (AC)_*n*_, D1S2659 (CA)_*n*_, and D1S212(CA)_*n*_ locate distal to *SERPINC1*.

For the determination of the age of ATBp3, genomic DNA from 36 unrelated index subjects with ATBp3 mutation and their family members out of the large AT-deficient group were haplotyped for alleles at eight STRs; seven dinucleotide and one tetranucleotide repeats; heterozygosity >0.7, namely D1S212 (4.92 ΔcM), D1S2659 (3.27 ΔcM), D1S218 (0.73 ΔcM), D1S2790 (1 ΔcM), D1S1165 (2.43 ΔcM), D1S2815 (2.53 ΔcM), D1S196 (7.35 ΔcM), and D1S460 (54.8 ΔcM) flanking the disease locus. Distances of the markers from ATBp3 are given in the brackets (Figure 1). The fragments containing the STRs were amplified by PCR, and the amplicons were tested for length polymorphism by capillary electrophoresis on an ABI3130 Genetic Analyzer (Thermo Fisher Scientific). Analysis of STR sequences was implemented by the GeneMapper v4.1 software (Life Technologies). Two hundred individuals out of the *n* = 1,000 Hungarian reference population as control subjects and *n* = 94 individuals out of the *n* = 1,185 Roma reference samples were also tested for these STR markers.

### Statistical Analysis and Computations

Kolmogorov-Smirnov test and Shapiro-Wilk test were performed to examine the normality of data distribution. Results of continuous variables were expressed as median and interquartile range. Differences between groups were analyzed by Student's *t*-test and ANOVA when normally distributed or by Mann-Whitney test and Kruskal-Wallis test in the case of non-normal distribution. Significance values were adjusted by the Bonferroni correction for multiple comparisons. Differences in category frequencies were evaluated by χ^2^-test. A *p*-value of 0.05 or less was considered to indicate statistical significance. All statistical analyses were performed using the Statistical Package for the Social Sciences (SPSS 23.0), Chicago IL, USA.

The age of an allele is the duration of time elapsed since it was created by a mutation (49). Age estimates can be made by different methods considering variations at marker loci and allele frequency. Linkage disequilibrium (LD) index values (δ) were calculated according to Bengtsson and Thomson (50). Genetic distances [recombination fraction (θ)] were obtained from physical distances (Genome Reference Consortium Human Build 38) between STRs and *SERPINC1* by applying a conversion factor of 1.17 cM/Mb derived from the Marshfield map (51). The age of ATBp3 [in generations (*g*)] was initially estimated by two moment methods. The first one is based on the algorithm of Risch et al., where *g* = logδ/*log*(1−θ) (52). The second method generates a Markov transition matrix (*K*), which gives the probability that, in a single generation, any one haplotype will be transformed into any other one. *K* is calculated as the weighted sum of matrices corresponding to the recombination (*R*), mutation (*M*), and no event occurring (*I*): *K* = θ*R* + μ*M* + (1−θ−μ)*I* (53). Under this model, it was possible to correct the decay of LD over generations for the mutation rate (μ) at marker loci (under the assumption of μ = 2.1 × 10^−3^ for D1S1165 and μ = 5.6 × 10^−4^ for dinucleotide-repeat STRs) (54). The number of generations that have passed since the foundation event was estimated by multiplying the state vector by *K* iteratively until the observed proportion of ancestral haplotypes on mutation-bearing chromosomes were reached. The number of times that *K* had been multiplied yielded the estimate of the age (*g*).

Besides simple parametric age estimators, we reanalyzed LD data in a Bayesian perspective using the Markov chain Monte Carlo method (55), implemented in the DMLE+ program, version 2.3 (available at dmle.org), and a second likelihood method (56), which estimates the distance (in generations) from probands sharing a common haplotype to the most recent common ancestor (MRCA) by the means of the ESTIAGE software. By DMLE+, the posterior probability distribution (*P*) of the ATBp3 (c.391C>T) mutation's age was inferred assuming a proportion of mutation-bearing chromosomes in our sample (*f* = 0.012) that conservatively represents the expected allele frequency in the Hungarian population and allows obtaining good convergence of *P*. The population growth parameter (*r* = 0.079) was calculated by the equation *T*1 = *T*0 × *eg* × *r*, in which T1 is the size of the actual population (9.8 million of inhabitants), T0 is the estimated size of the population at the time of the founder event (first half of the XVII century; 3.5 millions) and *g* is the number of generations passed since the founder event that had been estimated by the moment methods (57). The stepwise mutation model for STRs (mean μ = 10^−3^) was adopted to keep in consideration microsatellite instability while running ESTIAGE.

## Results

### Results of Genetic Epidemiology Studies

#### Age and Origin of the Type IIHBS Antithrombin Budapest 3 Mutation

As the founder effect of ATBp3 mutation was confirmed previously, we aimed to investigate the age and origin of the most recent common ancestor (MRCA) of the ATBp3 (c.391C>T) and to provide a plausible historical and demographic scenario in which the founder effect could have started. By investigating the *n* = 36 unrelated ATBp3 mutants and their family members (*n* = 70, altogether *n* = 106) a fully conserved ancestral haplotype: D1S212: 20; D1S2659: 11; D1S218: 24/25; D1S2790: 20; D1S1165: 13; D1S2815: 18; D1S196: 12; D1S460: 7 (where numbers after the markers represent the most frequent repeat number variations) was identified in 14 independent chromosomes (five independent chromosomes if only the index patients were taken into consideration, Supplementary Table 1). In addition, related haplotypes, likely derived from the ancestral one by either recombination or mutation at the flanking markers, were found in the remaining chromosomes. We analyzed the frequencies of repeat alleles associated with ATBp3 and compared them with the corresponding allele frequencies in 200 unrelated control Hungarian subjects recruited from the general Hungarian population who did not carry ATBp3 mutation. The most frequent haplotype in the Hungarian population for the investigated markers were as follows: D1S212: 20; D1S2659: 15; D1S218: 25; D1S2790: 21; D1S1165: 11; D1S2815: 18; D1S196: 12; D1S460: 7. As it is shown by LD index values obtained for the STR markers, the correlation between D1S218, D1S2790, D1S1165, and D1S2815 markers with ATBp3 allele was higher than in the case of markers D1S2659, D1S196, D1S212, and D1S460 (Table 1). The mean ± SD overall age estimate for the *SERPINC1* c.391C>T mutation, based on the LD data for the eight STRs, was 11.5 ± 5.47 *g* according to the first moment method (Table 1) and it was 11.8 ± 7.1 *g* according to the iterative procedure of the second one. The Markov chain Monte Carlo method provided similar estimation of 13 *g* (95% credible set (CS): 3–42 *g*), and the age estimation was 14 *g* (95% CS: 5–37 *g*) by the second likelihood method.

**Table 1:** Estimation of the age of the c.391C>T mutation in the *SERPINC1* gene by different methods.

**Marker**	**Distance**	**Repeat number variations**	**LD**	**MRCA age**		**Founder event of ATBp3 (date)**	
	**ΔcM**		**δ**	***g***	***y***	**Date**	**95% CS**
D1S212	4.92	20	0.326	11.5 ± 5.47	287 ± 137	1,703 ± 137	NA
D1S2659	3.27	11	0.628				
D1S218	0.73	24/25	0.945				
D1S2790	1.00	20	0.899				
D1S1165	2.43	13	0.741				
D1S2815	2.53	18	0.731				
D1S196	7.35	12	0.470				
D1S460	54.8	7	0.086				
All haplotypes	DMLE+			13	325	1,665	940–1,915
All haplotypes	ESTIAGE			14	350	1,640	1,065–1,865

*Distance (ΔcM, centiMorgan) is the genetic distance between the marker and the SERPINC1 locus on chromosome 1. Repeat number variations represent the most frequent repeat numbers in homozygous ATBp3 probands. Historical date of the founder event that has likely increased the frequency of the c.391C>T mutation in the Hungarian population, based on the MRCA age and a mean date of birth for mutation's carriers equal to 1990*.

*NA, not applicable; CS, credible set (Bayesian analog of confidence interval in classical frequentist statistics); LD, linkage disequilibrium between the allele and the SERPINC1 c.391C>T mutation; MRCA, most recent common ancestor of the c.391C>T-mutation-bearing chromosomes, whose age is expressed in generations (g) and years (y), assuming a mean value of 25 y/g; DMLE+, software implementing the Markov chain Monte Carlo algorithm to allow Bayesian estimation of the MRCA age; ESTIAGE, software implementing a likelihood-based method of inferring the MRCA age from multilocus haplotypes*.

Assuming an average of 25 years per generation and that the average birth year of the mutation carriers investigated is 1990, the present results suggest MRCA bearing the c.391C>T mutation back to middle of the XVII century. This dating points to the origin of the founder effect at a very turbulent period of the history of Hungary with settlement of different populations including Roma tribes.

#### Prevalence of Antithrombin Budapest 3 Mutation in the General Hungarian and Roma Population

As the age estimation of ATBp3 rose up the hypothesis of its Roma origin, we investigated the prevalence of this mutation in a large group of Roma individuals (*n* = 1,185) representing the Roma general population living in Northeast Hungary. The carrier frequency was 2.80% in this group, which was a considerably high as compared with our group representing the general Hungarian population (*n* = 1,000), where no ATBp3 carriers were found. To confirm our findings, second sets of samples were collected independently from the first recruitment. *N* = 402 Roma individuals and *n* = 450 apparently healthy individuals from the corresponding geographical area together with demographic and clinical data were involved. While, again, no ATBp3 was detected within the general Hungarian group, the carrier frequency of ATBp3 was 2.74% in the Roma group. In this latter group, 10 heterozygotes (age range 24–60 years) and one homozygote (age 28 years) were registered (Table 2). None of these patients suffered VTE, as yet, and two of them had MI in their case histories. However, some of them were very young at the time of data collection and did not present any cardiovascular risk factors. Notably, almost all Roma ATBp3 individuals had a BMI greater than 25 kg/m^2^.

**Table 2:** Demographic and clinical data of Roma carriers of antithrombin Budapest 3 collected from the confirmatory study.

**ID**	**Gender**	**Age (year)**	**ATBp3 genotype**	**ATE or VTE in case history**	**Presence of CV risk factors**
1	Male	24	Heterozygote	No	Obesity
2	Male	27	Heterozygote	No	No
3	Female	28	Homozygote	No	No
4	Female	35	Heterozygote	No	Obesity
5	Female	42	Heterozygote	No	Obesity, HLP
6	Female	53	Heterozygote	Yes (MI)	Obesity, DM, HT
7	Female	54	Heterozygote	No	No
8	Male	56	Heterozygote	No	Obesity, HT, HLP
9	Female	57	Heterozygote	No	DM, HT
10	Female	58	Heterozygote	Yea (MI)	Obesity, HT, HLP
11	Female	60	Heterozygote	No	Obesity, DM, HT

*The term CV (cardiovascular) risk factors cover hypertension (HT), diabetes mellitus (DM), hyperlipidemia (HLP), smoking, and obesity (BMI above 25 kg/m^2^)*.

**Figure 2:**
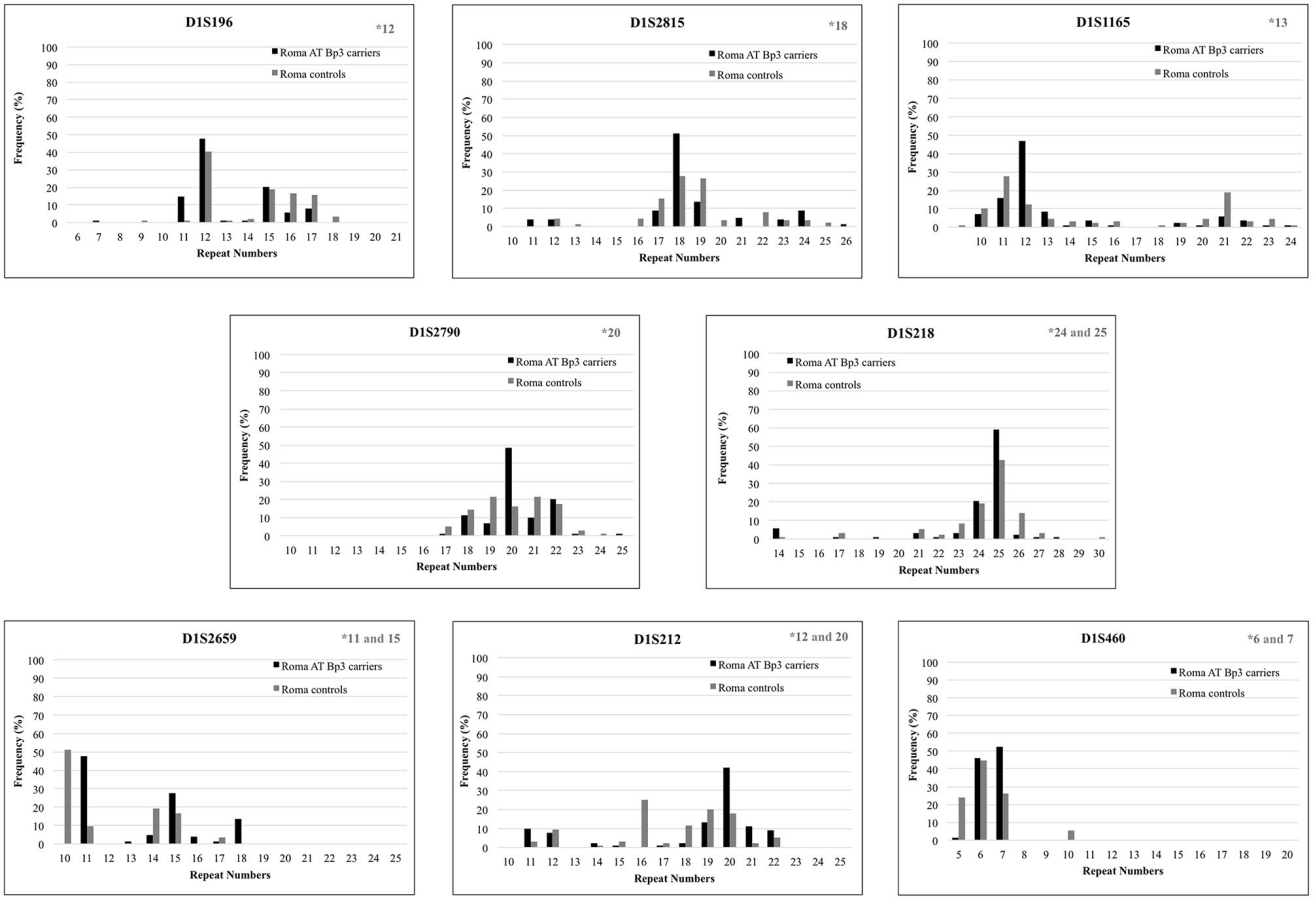
Repeat number variations of eight short tandem repeat markers in Roma ATBp3 carriers and in Roma controls. The most frequent repeat number variations detected at ATBp3 homozygous patients previously are marked in gray in the upper right corner of each graph.

To confirm the founder effect in the Roma population the same STR markers were analyzed in *n* = 44 ATBp3 carriers and in *n* = 50 non-carriers selected from the two Roma cohorts. The most frequent repeat number variations matched with the previously found distinctive repeat numbers (marked in gray in the upper right corner of Figure 2) except for markers D1S1165 and D1S212. As all but one Roma ATBp3 mutation carriers investigated for STR markers were heterozygous for the ATBp3 mutation, they also have a normal “C” allele, which might be associated with various repeat numbers. The most frequent haplotype in the Roma general population for the investigated markers were as follows: D1S212: 16; D1S2659: 15; D1S218: 25; D1S2790: 22; D1S1165: 12; D1S2815: 18; D1S196: 12; D1S460: 10. Other types of AT deficiency were not revealed within our Hungarian and Roma reference groups.

### Results of the Investigations on the Association Between Type IIHBS AT Deficiency and Thrombotic Diseases

#### Prevalence of Thrombosis Among Type IIHBS AT-Deficient Patients

Within our large cohort of AT-deficient patients (*n* = 435), the ratio of type IIHBS AT deficiency was extremely high; it was 75.4% (*n* = 328 type IIHBS AT-deficient patients, including *n* = 19 AT Basel, *n* = 30 AT Padua and *n* = 279 ATBp3 individuals). Most of them were index patients, only *n* = 85 individuals were found during family screening. As detailed clinical information was eligible in the case of *n* = 230 type IIHBS AT-deficient patients from the point of view of cardiovascular events, we took only them into consideration for further analysis. Current median age of our type IIHBS cohort was 34 years; however, ATBp3 homozygous patients were significantly younger due to their early diagnosis of AT deficiency because of early thrombotic episodes (Table 3). Accordingly, their age at the first thrombotic episode was also significantly lower as compared with others. There were more relatives registered in ATBp3 heterozygous group than in other groups, due to some positive cases found by family screening of ATBp3 homozygotes. The prevalence of thrombotic events, as expected, was significantly higher in the group of ATBp3 homozygotes as compared with any other groups. VT events (VTE) were registered in almost all patients with ATBp3 homozygosity (93%), while the frequency of VTE was below 50% in other groups. The frequency of provoked thrombosis (thrombosis during pregnancy or childbirth, trauma, surgery, oral anticoncipient use within 1 month of the thrombotic episode) was rather low in our study group; *n* = 2 patients in the AT Padua group, *n* = 2 patients in ATBp3 homozygotes group, and *n* = 11 among ATBp3 heterozygotes. There was only one patient with unprovoked VTE among AT Basel patients. VTE was recurrent in one-third of the patients in average, and there were no significant differences among the different groups. The highest number for recurrent VTE was 7, registered in an ATBp3 homozygous patient. Three to six episodes of VTE were registered in other ATBp3 homozygotes and in some ATBp3 heterozygotes, while a maximum of two VTE episodes were described in AT Padua. Isolated PE was found in eight ATBp3 homozygotes, in three ATBp3 heterozygotes, and in three AT Padua patients, and one ATBp3 homozygous patient suffered from three episodes of PE. We investigated whether carriership of FVL influences the clinical phenotype in ATBp3 patients. In ATBp3 homozygotes, only FVL heterozygous patients (*n* = 4) were registered. Clinical phenotype of ATBp3 homozygosity seemed severe enough not to be influenced by the presence or absence of a heterozygous FVL as clinical presentation of thrombosis in terms of the number of thrombotic episodes and the age at first symptoms were similar in patients with FVL and in wild type patients. In the group of ATBp3 heterozygotes, five patients were registered as FVL homozygotes. Among them, only two patients had thrombotic episodes in their case histories and they were relatively old, one of them was 47 years old at the time of his VT, the other patient had one episode of PE at the age of 65 years. Others had no thrombosis, so far; however, they are still young, below the age of 20 years. If FVL heterozygotes and wild-type patients were compared within the group of ATBp3 heterozygotes, no significant differences were found in terms of the frequency of patients with thrombotic episodes (around 50%) and types of thrombosis (frequency of VT 44 and 45% for wild type and heterozygotes, respectively, PE 6% in both groups, ATE 10 and 6% for wild type and heterozygotes, respectively); however, heterozygotes were a bit younger at the time of the first symptoms. (Median age of wild-type and FVL heterozygous patients at first thrombosis was 34 and 27 years, respectively, *p* = 0.05). FVL heterozygotes presented recurrent thrombosis more often than wild-type patients (frequency of recurrence for wild type and heterozygotes were 16 and 40%, respectively, *p* = 0.04). To conclude these findings, carriership of FVL in ATBp3 heterozygous subjects may shift the age at the first thrombotic episode to a lower range and it may have an impact on the severity (if it is characterized by recurrence).

**Table 3:** Clinical and laboratory characteristics of the type IIHBS antithrombin-deficient cohort.

	**All (*n* = 230)**	**AT Basel(*n* = 9)**	**AT Padua (*n* = 26)**	**AT Budapest3 HoZ(*n* = 44)**	**AT Budapest3 HeZ (*n* = 151)**	***p-*value**
Current age years^a^ (median; min–max); IQR	34 (2–76);26	51 (28–65);23	42 (18–70);23	24 (2–69);21	34 (2–74);26	<0.001
Male/female (*n*)	93/137	3/6	6/20	22/22	62/89	0.162
Index pts/relatives^b^ (*n*)	162/68	7/2	22/4	36/8	97/54	0.041
Any thrombotic event Y/N^c^ (*n*)	131/99	5/4	12/14	41/3	73/78	<0.001
Age at first thrombotic event years^d^ (median; min–max); IQR	26 (0–68);24	42 (15–51);34	50 (22–66);17	15 (0–48);16	33 (0–68);19	<0.001
VT %^e^	52	11	38	93	44	<0.001
Recurrent VT % within VT patients	35	0	29	38	34	0.831
ATE %^f^	8	44	8	5	6.6	<0.001
Ratio of FVL carriers HeZ/HoZ %	21/2	0	24/0	9/0	25/3	0.261
Ratio of FII 20210A carriers HeZ %	3.9	0	7.6	5.9	2.9	0.738
Antithrombin activity %^f^ (median; min–max); IQR	56 (9–78);11	57 (44–74);11	57.5 (40–74);12	17 (9–59);10	57 (37–78);8	<0.001
Antithrombin progressive activity %^g^(median; min–max); IQR	85 (56–126);15	100 (73–117);17	105 (73–126);23	73 (56–100);11	85 (60–114);10	<0.001
Antithrombin antigen g/L^g^(median; min-max); IQR	0.25 (0.13–0.35);0.05	0.3 (0.25–0.32);0.03	0.3 (0.24–0.35);0.04	0.2 (0.13–0.28);0.03	0.25 (0.17–0.31);0.04	<0.001

*Isolated PE was diagnosed in 14 cases (n = 3 AT Padua, n = 3 ATBp3 HoZ, n = 8 ATBp3 HeZ)*.

*IQR, interquartile range; HeZ, heterozygote; HoZ, homozygote*.

a *Current age of ATBp3 homozygotes is significantly lower than current age of other groups (p < 0.001 as compared with AT Basel and Padua and p = 0.006 as compared with ATBp3 HeZ); current age of ATBp3 heterozygotes is significantly lower than current age of AT Padua (p = 0.014)*.

b *Ratio of index patients vs. relatives was lower in ATBp3 HeZ than in other groups (ATBp3 HoZ vs. ATBp3 HeZ p = 0.028, AT Padua vs. ATBp3 HeZ p = 0.044, AT Basel vs. ATBp3 HeZ p = 0.496)*.

c *Prevalence of any thrombotic events was the highest in ATBp3 homozygous group (AT Basel vs. ATBp3 HoZ p = 0.012, AT Padua vs. ATBp3 HoZ p < 0.001, ATBp3 HeZ vs ATBp3 HoZ p < 0.001)*.

d *Age at first thrombotic event of ATBp3 homozygotes is significantly lower than that of other groups (p < 0.001 as compared with ATBp3 HeZ and AT Padua and p = 0.005 as compared with AT Basel). Age at first thrombotic event of ATBp3 heterozygotes is significantly lower than that of AT Padua (p = 0.002)*.

e *Prevalence of VT events was the highest in ATBp3 homozygous group (AT Basel vs. ATBp3 HoZ p < 0.001, AT Padua vs. ATBp3 HoZ p < 0.001, ATBp3 HeZ vs ATBp3 HoZ p < 0.001)*.

f *Antithrombin heparin cofactor and progressive activity and AT antigen of ATBp3 homozygotes are significantly lower than AT activity and antigen of other groups (p < 0.001 in all comparisons)*.

g *Progressive antithrombin activity and AT antigen of ATBp3 heterozygotes are significantly lower than AT activity and antigen of AT Basel and Padua (p = 0.004 and p < 0.001 for AT progressive activity, respectively, and p < 0.001 for AT antigen for both)*.

The number of ATE was much less than that of VTE; however, it was not negligible. It was remarkable that AT Basel, patients showed a higher frequency of ATE, as compared with others; four patients out of the 9 suffered from ATE without having VTE in their case histories and in two of them the ATE was recurrent (Table 4). They were all young at their first ATE. A young female patient with ATBp3 homozygous mutation suffered ischemic stroke at the age of 40 years and formerly she had three episodes of VTE, the first was registered at the age of 15. Another young female with two VTE in her case history suffered from ischemic stroke at the age of 27 years without reporting any risk factors for cardiovascular diseases. It is interesting that both patients have hypoplasia of the vena cava as diagnosed by computer tomography. Both patients were put on life-long anticoagulant treatment after their second thrombotic episodes by warfarin. Among ATBp3 heterozygotes, *n* = 10 patients were registered with ATE. Half of them had VTE in their case histories and two of them suffered from more than one episode of ATE. The youngest patient is a currently 11-year-old boy who suffered his first episode of ischemic stroke at the age of 2 followed by two additional episodes until now. His case is however different from others, since—although there was no embolic source confirmed in the background—due to the presence of atrial and ventricular septal defects, we cannot exclude the venous origin of vascular occlusion. The eldest patient with ATE was still relatively young; she was 48 years old at the time of MI.

**Table 4:** Major clinical characteristics of type IIHBS antithrombin deficient patients with arterial thrombotic disease.

**Patient ID**	**AT deficiency type**	**Gender**	**VT No. (age at first event in years)**	**ATE (age at event)**	**Presence of other thrombophilia**	**Presence of CV risk factors**	**Other clinical conditions**
1	AT Basel	Male	0	MI (15)	0	HLP	0
2	AT Basel	Female	0	MI (50)	0	ND	0
3	AT Basel	Male	0	MI (42), stroke (45)	0	0	0
4	AT Basel	Female	0	Stroke (49)	0	0	0
5	AT Padua	Male	0	Stroke (43)	0	ND	0
6	AT Padua	Female	0	Stroke (40)	0	0	0
7	AT Bp3 HoZ	Female	3 (15)	Stroke (40)	0	0	v. cava inferior hypoplasia
8	AT Bp3 HoZ	Female	2 (17)	Stroke (27)	0	0	v. cava inferior hypoplasia
9	AT Bp3 HeZ	Female	1 (29)	Stroke (39)	0	0	0
10	AT Bp3 HeZ	Male	0	Stroke (14)	0	HLP, Hcy	0
11	AT Bp3 HeZ	Female	1 (23)	Stroke (23)	0	0	0
12	AT Bp3 HeZ	Male	2 (27)	Stroke (19), MI (24)	FVL HeZ	Smoking	0
13	AT Bp3 HeZ	Male	0	Stroke (2), +2 stroke	0	0	ASD and VSD
14	AT Bp3 HeZ	Male	0	MI (47)	FVL HeZ	Smoking, HLP	0
15	AT Bp3 HeZ	Female	1 (47)	MI (38)	0	HT, obesity	Hypothyreosis
16	AT Bp3 HeZ	Female	1 (37)	MI (48)	0	HT	Aorta stenosis
17	AT Bp3 HeZ	Female	0	Stroke (18)	FVL HeZ	0	0
18	AT Bp3 HeZ	Female	0	MI (38)	0	Smoking	0

*The term CV risk factors cover hypertension (HT), diabetes mellitus, hyperlipidemia (HLP), hyperhomocysteinemia (Hcy), smoking and obesity (BMI above 25 kg/m^2^)*.

*No., number; HeZ, heterozygote; HoZ, homozygote; ASD, atrial septal defect; VSD, ventricular septal defect; ND, no data*.

#### Prevalence of Type IIHBS Mutations in Young Patients With Myocardial Infarction

As ATE was registered in our type IIHBS AT-deficient cohort, even in ATBp3 heterozygotes and patients with these thrombotic symptoms were relatively young at the onset of the disease, we investigated the prevalence of type IIHBS AT deficiency among young individuals with MI. As the prevalence of MI is low below 40 years of age, we could recruit *n* = 119 individuals with MI below the age of 40 in their case histories and *n* = 101 age-matched individuals without MI (clinical controls, CC). Recruitment lasted for 5 years. Only those individuals in both groups, who underwent cardiac catheterization upon admission, were included. After informed consent *n* = 92 MI and *n* = 74 CC patients were eligible for the study (Table 5). Concerning classical thrombophilia risk factors, almost 10% of MI patients were carriers of the FVL; however, based on the allele frequency data of the general population in Hungary—this is not surprising and it was not different from the CC group. Prevalence data of FII20210A carriers were also similar in the two groups. While no protein C or protein S deficiencies were found among these patients, there were three young MI patients with type IIHBS AT deficiency in our study group. Two of them were heterozygous carriers of the ATBp3 mutation, while one patient was a heterozygous carrier of the AT Basel mutation; all patients were males. There were no AT-deficient individuals in the CC group. Among the three patients with type IIHBS mutations in the MI group, the young patient with AT Basel suffered from MI (occlusion of LAD) at the age of 16. Two years after the coronary stent implantation, he had ischemic stroke (a. cerebri media). He was put on anticoagulant therapy (first with warfarin, then with apixaban 2 × 5 mg) combined with aspirin (100 mg/day). At the age of 26, while on dual pathway inhibition, he suffered inferior STEMI with right coronary artery (RCA) occlusion and PCI was performed. He has no other known thrombophilia and he is also wild type for FVL and FII20210. He has no classical cardiovascular risk factors, except for an elevated Lp(a) above 800 mg/L. The two patients with ATBp3 suffered from MI at the age of 39 and 40. The first patient had inferior STEMI with RCA occlusion; the second patient had STEMI with LAD occlusion. Both patients underwent coronary stent implantation. Due to in-stent restenosis, the second patient had re-coronarography and drug-eluting stent implantation. Both patients were put on clopidogrel therapy. None of them suffered from VTE until data analysis. None of them had other thrombophilia; however, they had hyperlipidemia with normal lipid parameters on adequate therapy, and they were active smokers with pack-years of 25 and 20, respectively. The 40-year-old patient was obese, with a BMI of 40, and he had type 2 diabetes mellitus with normal glucose values on therapy.

**Table 5:** Clinical and laboratory characteristics of the young MI and venous thrombosis populations and their healthy controls.

	**HC (*n* = 215)**	**CC(*n* = 74)**	**MI (*n* = 92)**	**VTE(*n* = 110)**	***p*-value (MI vs. VTE)**	***p*-value(MI vs. CC)**	***p*-value (VTE vs. HC)**
Age years (median; min–max); IQR	27 (18–40);12	35 (20–40);6	36 (14–40);4	30.5 (17–40);11	<0.001	0.129	0.076
Male/female (*n*)	87/128	47/27	70/22	57/53	0.001	0.089	0.059
Diabetes mellitus (%)	0	12.0	14.0	4.0	0.010	0.819	0.015
Active smokers (%)	25.0	21.0	60.0	20.0	<0.001	<0.001	0.070
Ex-smokers %	9.5	20.8	33.3	18.0	<0.001	<0.001	0.084
ATE+VTE %	0	0	4.3	1.8	0.012	NA	NA
Positive family history ATE or VTE (%)	45.0	60.0	74.0	62.0	<0.001	0.060	<0.001
Hypertension (%)	7.0	51.0	43.0	12.0	<0.001	0.350	0.149
Hyperlipidemia (%)	0	39.0	86.0	0	NA	<0.001	NA
BMI kg/m^2^ (median; min–max); IQR	23.2 (16.3–41.4);6.39	28.0 (19.0–45.0);10.0	28.0 (17.0–46.0);6.0	28.7 (18.9–52.7);7.32	0.221	0.586	<0.001
Type IIHBS AT deficiency %	0	0	3.3	1.8	0.469	NA	NA
FVL carriers HeZ/HoZ %	8/0	9.6/0	9.4/0	35.5/10.9	<0.001	0.970	<0.001
FII 20210A carriers HeZ %	3.3	1.4	2.3	6.3	0.304	0.652	0.251
Antithrombin activity % (median; min–max); IQR	106 (80–130);14	105 (87–135);11	112 (57–135);12	104 (50–136);15	<0.001	<0.001	0.042
AT, PC, and PS deficiency %	0	0	3.3	5.5^*^	0.469	NA	NA

*Positive family history was defined as MI, stroke, or non-traumatic lower limb amputation in first-degree relatives*.

*NA, not applicable; IQR, interquartile range; HeZ, heterozygote; HoZ, homozygote*.

*Only PC activity showed normal distribution. Reference intervals for antithrombin activity is 80–120%*.

* *There were two patients with genetically confirmed type IIHBS antithrombin deficiency (ATBp3 heterozygotes) and protein C deficiency, one patient with protein S deficiency, and one patient with type I antithrombin deficiency in the venous thrombosis group*.

#### Prevalence of Type IIHBS Mutations in Young Patients With Venous Thromboembolic Disease

As a comparison with our young MI patients, we investigated consecutive patients suffering from VTE below the age of 40 (*n* = 110) recruited from our large cohort of 400 VTE patients. In this group, *n* = 26 (24%) patients were registered with provoked thrombosis (*n* = 7 childbirth, *n* = 3 immobility, *n* = 11 oral anticoncipients, *n* = 5 trauma). All other thrombotic episodes were spontaneous. Healthy controls (*n* = 215) below the age of 40 upon recruitment and selected from our healthy reference population (i.e., out of the *n* = 450 healthy Hungarian samples) served as a general reference for all patients' groups (Table 5). The representation of females was higher in the VTE group as compared with our MI patients. There were huge differences in the prevalence of classical cardiovascular risk factors between VTE and MI groups, while BMI values did not differ. While 4.3% of MI patients also had VTE in their case histories, only 1.8% of VTE patients registered with arterial events (*p* = 0.012). Among classical thrombophilia risk factors, the allele frequency of FVL was significantly higher in the VTE group (more than 45% of the patients were carriers) and not only heterozygotes but also homozygotes were found. The frequency of FII20210A carriers was only slightly higher in the VT group. Type IIHBS deficiency was registered in two VTE patients (both ATBp3 heterozygotes); moreover, one type I AT deficiency, two PC deficiencies, and one PS deficiency were confirmed by genetic testing. As being exclusion criteria, the healthy reference group was free of diabetes mellitus and hyperlipidemia and the representation of active smokers was comparable with the CC and VTE groups. The median BMI was lower than that of the other groups. Allele frequency values of FVL and FII20210 were similar to that of CC and MI groups and no AT, PC or PS deficiency was explored. The carrier frequency of type IIHBS AT deficiency was also zero.

## Discussion

In this study, we investigated the age and origin of the founder ATBp3 mutation and strengthen the hypothesis of its Roma origin. Both Bayesian estimates were consistent each other and with the MRCA age obtained by the moment methods. All methods suggested that ATBp3 mutation founding was ~350–400 years ago. The upper limit of the CS age interval was higher in the case of Bayesian methods than the corresponding interval suggested by the mean ± SD value obtained from parametric analysis of LD decay over generations by the two moment methods; this difference might be due to the relatively small sample size that we could collect for analysis; however, this sample size was the maximum feasible in case of such a rare disease. We observed that homozygous ATBp3 mutation carriers had a bi-allelic distribution of D1S218 marker. This marker shows the least distance from ATBp3. If no recombination is assumed at this point since the mutation occurred, it might happen that the founder event occurred parallel in two alleles carrying two different D1S218 STR variants. It is a more possible explanation of this phenomenon; however, that the mutation is young enough to occur only one recombination event at position D1S218 since the foundation event. Upon confronting the MRCA age with the major population events that occurred at different periods of the Hungarian history, a very turbulent period was recognized. It was characterized by the Fifteen-Year War (1592–1606) then the Thirty-Year War (1618–1648), plus four plague epidemics (1620–1627, 1632–1634, 1643–1645, and 1660–1665), and a variola outbreak (57). These dramatic events brought famine and other calamities to the entire region. In some areas, a massive population decline occurred; however, at the same time, the arrival of Serbs in regions under Ottoman rule and of Romanians, Ruthenians, Vlachs, and Romanies in Royal Hungary, might created population bottleneck, through which a pre-existent or a *de novo SERPINC1* c.391C>T mutation could be introduced into the Hungarian population and became fixed because of strong genetic drift. A rapid population growth afterwards could strengthen a founder effect for c.391C>T. It was mentioned in historical literature that major immigration ways of Roma people to Hungary were from Serbia and from Romania and Transylvania, and during the XVII Century, the Roma population in Hungary had grown significantly due to immigration (58). As no data of official census is available from that century, the exact number of Roma immigrants and population size in XVII Century is not known. Until the end of the XVIII century when the first official census was carried out, however, the frequency of Roma in Hungary reached 1% of total population. In conclusion, the estimated age of the *SERPINC1* c.391C>T mutation, the geographic distribution of families with ATBp3, and the history of the modern Hungarian population are consistent with the hypothesis that the mutation originated (or was originally introduced) and expanded in the later Ottoman period and Royal Hungary. Almost all patients with ATBp3 mutation have been reported so far are of Central Eastern European origin, and this is consistent with the proposed origin of the founder effect (8, 59–62). The considerable carrier frequency (around 3%) in such a rare disease that was found in the Roma general population and the confirmation of the founder effect of the ATBp3 mutation further strengthens the hypothesis that the mutation is of Roma origin, or at least became more prevalent in that population due to high frequency of consanguinity and inbreeding (63). We do not have clear explanation on whether the ATBp3 mutation provided an advantage for carriers; only speculations can be made. Similarly to that of an advantage of being FVL carrier, we may surmise that being heterozygous for ATBp3 mutation prevented high blood loss upon childbirth and injuries in the Roma population with nomadic lifestyle. Since homozygosity exerts an extremely high thrombotic risk, only heterozygosity might have an advantage over wild genotype in this context.

As it was demonstrated earlier, the Roma population is vulnerable from the point of view of cardiovascular diseases (41, 43). They tainted with several environmental and genetic risk factors making them more sensitive to early thrombosis development, including ATE. In addition to obesity and other risk factors, Roma people are often subjected to air pollution living in peripheral, industrial areas (58). Identification of individuals with more severe genetic load is of high importance to offer them adequate preventive and treatment strategies. Since we demonstrated here, that Roma people may be carriers of the type IIHBS ATBp3 mutation with a considerable frequency, the number of ATBp3 AT-deficient individuals among the 700,000–800,000 Roma people living in Hungary according to recent estimations is ~24,000, which number may be even higher in the Central European region, where the number of Roma inhabitants exceeds 10 million (64). Besides its genetic epidemiological value, the identification of a vulnerable population loaded with the ATBp3 mutation and a documented clinical history will be extremely useful in the search for disease modifying loci within c.391C>T-carrying families. As FVL is also prevalent in the Roma population, the chance of having combined thrombophilia by carrying both FVL and ATBp3 is not negligible. Based on our results it is recommended to include the investigation of ATBp3 AT deficiency as part of thrombosis risk-stratification in Roma individuals.

It is of outmost importance to identify other vulnerable individuals and populations from the point of view of disease control and prevention. Here, we attempted to examine the association of ATBp3 and other type IIHBS mutations with cardiovascular diseases including not only venous but also arterial thrombotic events, as well. The question is interesting from several aspects. It was hypothesized earlier by us and by others that AT deficiency was a rather heterogeneous disease concerning its clinical and laboratory consequences (7, 13, 14). Even, within type IIHBS deficiency, heterogeneity was observed in the clinical symptoms, onset of diseases and risk of recurrent thrombotic events. It is now evident, as supported by cohort studies and case reports, that ATBp3 homozygosity is one of the most severe thrombophilia (9, 10, 13, 65) and is evidently associated with severe VTE. ATBp3 heterozygous state is less severe; however, its association with VTE is also significant. Recently, reports on association of ATBp3 and other type IIHBS AT deficiencies with ATE have been published; however, as hereditary AT belongs to the group of rare diseases, well-designed, large clinical studies with high statistical power cannot be carried out (6, 10, 11, 61). Multicenter studies may overcome this problem; however, they are likely to be subjected to bias due to heterogeneous ethnicity. Until having more robust data (hopefully from the International AT deficiency registry, which has been started most recently under the umbrella of the Scientific and Standardization Committee on Plasma Coagulation Inhibitors of the ISTH), each piece of evidence is helpful for clinicians facing AT-deficient cases. In well-described case reports, most of the AT-deficient patients were young at the development of their first MI or stroke suggesting that AT deficiency is severe enough to lead to early onset thrombotic complications even at the “arterial side” (66–71). As being a rare disorder, however, only a few publications have been released in which the role of AT deficiency was investigated in ATE, as yet. In observational cohort studies recruiting unselected MI or stroke patients, only a small proportion, if any are expected to carry AT mutations (72–74). Clinically useful conclusions, therefore, are hardly drawn from these studies. Investigation of selected populations may have more rationale and results from these studies may be more relevant for the specific source population from which the sample is selected. As an example to this, AT Cambridge II (p.Ala416Ser according to HGVS nomenclature), which is a relatively frequent mutation in the Spanish population associated with increased risk (OR 5.66; 95% CI 1.53, 20.88) of MI in a large sample (*n* = 1,224 MI patients and *n* = 1,649 controls) suggesting AT deficiency as an independent risk factor for MI (75). Another approach for investigating the association of AT deficiency with cardiovascular diseases is to conduct cohort studies in genetically confirmed AT-deficient patients' groups. According to the recent results of such studies, the prevalence of ATE was between 4.8 and 19%, and these events associated more often with type IIHBS AT deficiency than with other types (6, 7, 10, 11).

Since we have one of the largest type IIHBS AT-deficient cohort investigating the prevalence of ATE in different subtypes of type IIHBS AT deficiency, especially with ATBp3, seems feasible. ATE was most prevalent in AT Basel patients and almost all with this genotype have been suffered from at least one ATE episode until the time of data collection without having any VTE. It is to be noted, however, that the sample size of both AT Basel and Padua is much lower than that of ATBp3 in our population to draw a strong conclusion, although our findings are well-correlated with other reports (6, 10). In case of ATBp3, the frequency of ATE was higher in the heterozygote group as compared with homozygotes. The probable explanation for this is the lower age of ATBp3 homozygotes. They were twice younger than heterozygotes upon first hospitalization and suffered severe VTE as first thrombosis. As a consequence of early diagnosis of AT deficiency, they were immediately put on long-term anticoagulation and were warned to avoid any modifiable cardiovascular risk factors. So, according to our experience, ATBp3 homozygotes are rescued from the AT-deficient population before the development of arterial events. ATBp3 heterozygosity is another issue. The ratio of ATE was obviously lower as compared with VTE in our cohort; however, they were all young at the time of MI or stroke development. In eight out of 10 patients, there was no VTE at all in their histories, or at least ATE preceded the venous thrombotic episodes. At least one modifiable cardiovascular risk factor was registered in their cases drawing the attention to the possible combined effect of AT deficiency and environmental risk factors in the early development of ATE. Cardioembolic (or thromboembolic) origin of stroke was excluded in all but one case; patient 13 in Table 4 had both atrial and ventricular septal defects, which were closed at the age of 2 years; however, he suffered two more episodes of stroke afterwards, before the age of 11.

As ATE developed at a young age in our AT-deficient cohort, we aimed to investigate the effect of type IIHBS AT deficiency on ATE development in selected populations consisted of non-related young MI and VTE patients. The ratio of type IIHBS AT deficiency in MI patients was comparable with that of VTE patients, although two major differences were observed. MI patients had more classical cardiovascular risk factors; especially, the frequency of smoking and hyperlipidemia was very high, while the ratio of additional thrombophilia, especially FVL mutation, as expected, was significantly higher in the VTE group. In conclusion, the presence of type IIHBS AT deficiency is not negligible in young individuals suffering from any types of thrombosis, and searching for AT Basel or ATBp3 in such selected populations, especially, in regions, where these mutations are prevalent, may be beneficial.

Our study has some limitations. As a single-center study, we could not reach large sample size, thus high statistical power in young MI and VTE groups for investigating ATBp3 effect. However, the prevalence of ATE and VTE at a young age is generally low, and establishment of such a strict cut-off value for age (40 years) among inclusion criteria makes recruitment difficult. Moreover, as AT deficiency itself is a rare disease, especially the number of homozygous ATBp3 carriers in the ATE study was small. This limits the impact of our observations. We believe, that by the demonstration of the presence of ATBp3 (and also AT Basel) even in these relatively small groups of ATE (and VTE) patients, we could draw the attention to consider type IIHBS AT deficiency in the background of not only VTE but also ATE, especially in selected populations as young patients without advanced atherosclerosis. Another limitation is the uncertainty of ethnicity in the different patients' groups. As being a sensitive issue, Roma ethnicity is not registered upon hospitalization or patient recruitment except for those epidemiology studies, where directly Roma population is involved. By demonstrating the high prevalence of ATBp3—as compared with its rare disease status—in the Roma general population, we suggest that in regions, where the size of Roma minority is significant, the chance of diagnosing ATBp3 AT deficiency is higher. Collecting data in larger studies will help in clarifying the role of ATBp3 AT deficiency in different types of thrombosis and the risk conferred by the combination of this thrombophilia with other genetic and environmental cardiovascular risk factors in the development of these diseases.

## Data Availability Statement

The raw data supporting the conclusions of this article are available upon request from the corresponding author (ZB).

## Ethics Statement

The studies involving human participants were reviewed and approved by Committee of the Hungarian Scientific Council on Health and Ethical Committee of University of Debrecen, Hungary. Written informed consent to participate in this study was provided by the participants' legal guardian/next of kin.

## Author Contributions

ZB designed the research, was involved in all aspects of the study, evaluated results, and wrote the manuscript. RG was involved in all aspects of the study, analyzed results, and took part in writing the manuscript. SF was involved in recruiting Roma individuals and in sample collection. MS performed genetic analysis and data evaluation of founder effect. TM recruited patients with venous thrombosis. LB recruited myocardial infarction patients. ZM and ZS performed genetic investigations. RÁ was involved in designing and implementing Roma surveys, data analysis, and manuscript preparation. All authors reviewed the manuscript before submission.

## Conflict of Interest

The authors declare that the research was conducted in the absence of any commercial or financial relationships that could be construed as a potential conflict of interest.
