# Supplementary material for: Age and Origin of the Founder Antithrombin Budapest 3 (p.Leu131Phe) Mutation; Its High Prevalence in the Roma Population and Its Association With Cardiovascular Diseases
Source: Front Cardiovasc Med. 2021 Feb 5;7:617711. doi: 10.3389/fcvm.2020.617711 (PMC7892435; doi:10.3389/fcvm.2020.617711)
Supplement: Supplementary file 1 [file Table_1.DOCX]

**Supplementary Table 1.** Short tandem repeat variations in index patients with Antithrombin Budapest 3 mutation.

| **ID** | **Birth date (year)** | **AT Bp3 genotype** | **D1S212** | **D1S2659** | **D1S218** | **D1S2790** | **D1S1165** | **D1S2815** | **D1S196** | **D1S460** |
| --- | --- | --- | --- | --- | --- | --- | --- | --- | --- | --- |
| 1 | 1979 | mut/mut | 110 / 112 | 202 / 216 | 357 / 359 | 247 / 247 | 407 / 407 | 296 / 355 | 271 / 271 | 162 / 162 |
| 2 | 1974 | mut/wt | 94 / 112 | 202 / 210 | 359 / 359 | 249 / 253 | 400 / 404 | 355 / 355 | 270 / 270 | 162 / 162 |
| 3 | 1995 | mut/mut | 95 / 112 | 210 / 210 | 357 / 359 | 246 / 249 | 396 / 409 | 355 / 365 | 277 / 281 | 164 / 164 |
| 4 | 1996 | mut/wt | 94 / 112 | 202 / 216 | 359 / 365 | 247 / 249 | 392 / 405 | 355 / 365 | 270 / 270 | 164 / 164 |
| 5 | 2009 | mut/mut | 95 / 110 | 202 / 202 | 357 / 359 | 249 / 249 | 405 / 405 | 355 / 355 | 270 / 281 | 164 / 164 |
| 6 | 1993 | mut/mut | 95 / 112 | 210 / 216 | 357 / 359 | 249 / 249 | 405 / 405 | 355 / 355 | 270 / 281 | 162 / 165 |
| 7 | 1968 | mut/mut | 97 / 112 | 202 / 202 | 357 / 359 | 249 / 249 | 405 / 405 | 355 / 355 | 270 / 270 | 162 / 164 |
| 8 | 1973 | mut/mut | 95 / 112 | 203 / 203 | 357 / 359 | 249 / 249 | 405 / 405 | 355 / 355 | 270 / 277 | 165 / 165 |
| 9 | 1946 | mut/mut | 94 / 112 | 202 / 210 | 357 / 359 | 249 / 249 | 404 / 450 | 343 / 356 | 270 / 281 | 165 / 165 |
| 10 | 1995 | mut/mut | 96 / 108 | 210 / 212 | 357 / 359 | 249 / 249 | 405 / 409 | 355 / 365 | 277 / 281 | 165 / 165 |
| 11 | 1991 | mut/mut | 96 / 108 | 202 / 202 | 357 / 359 | 249 / 249 | 405 / 405 | 355 / 355 | 270 / 279 | 164 / 164 |
| 12 | 1992 | mut/mut | 95 / 112 | 202 / 210 | 357 / 359 | 249 / 249 | 405 / 405 | 355 / 355 | 270 / 277 | 162 / 165 |
| 13 | 2011 | mut/mut | 97 / 108 | 202 / 210 | 357 / 359 | 249 / 249 | 404 / 404 | 355 / 355 | 271 / 271 | 163 / 163 |
| 14 | 1995 | mut/mut | 94 / 112 | 202 / 210 | 357 / 359 | 249 / 249 | 405 / 405 | 356 / 356 | 271 / 283 | 164 / 164 |
| 15 | 1985 | mut/mut | 94 / 112 | 202 / 210 | 359 / 359 | 249 / 253 | 404 / 465 | 355 / 361 | 270 / 270 | 162 / 162 |
| 16 | 1998 | mut/mut | 94 / 112 | 202 / 202 | 357 / 359 | 249 / 249 | 404 / 404 | 355 / 355 | 271 / 279 | 162 / 162 |
| 17 | 1967 | mut/wt | 112 / 115 | 202 / 216 | 359 / 359 | 249 / 249 | 405 / 442 | 355 / 355 | 279 / 281 | 164 / 164 |
| 18 | 1997 | mut/mut | 96 / 110 | 202 / 210 | 359 / 359 | 247 / 247 | 405 / 437 | 355 / 362 | 270 / 270 | 164 / 164 |
| 19 | 2002 | mut/wt | 110 / 116 | 202 / 216 | 351 / 359 | 249 / 253 | 438 / 438 | 343 / 369 | 270 / 277 | 164 / 164 |
| 20 | 1995 | mut/mut | 97 / 112 | 202 / 202 | 357 / 359 | 249 / 249 | 405 / 405 | 355 / 355 | 271 / 277 | 165 / 165 |
| 21 | 1992 | mut/mut | 96 / 112 | 210 / 216 | 357 / 359 | 249 / 249 | 404 / 442 | 355 / 355 | 277 / 281 | 164 / 164 |
| 22 | 1995 | mut/wt | 94 / 110 | 202 / 210 | ND | 247 / 247 | 404 / 441 | 352 / 355 | ND | 162 / 164 |
| 23 | 1985 | mut/wt | 94 / 112 | 202 / 210 | ND | 249 / 249 | 400 / 404 | 355 / 357 | ND | 164 / 164 |
| 24 | 1994 | mut/wt | 114 / 114 | 202 / 210 | 358 / 360 | 249 / 253 | 396 / 405 | 355 / 355 | 271 / 282 | 164 / 164 |
| 25 | 2004 | mut/mut | 95 / 112 | 202 / 207 | 358 / 360 | 249 / 249 | 405 / 405 | 355 / 355 | 270 / 270 | 162 / 162 |
| 26 | 1950 | mut/wt | 96 / 114 | 204 / 212 | 352 / 360 | 246 / 249 | 443 / 451 | 352 / 357 | 271 / 283 | 164 / 164 |
| 27 | 1996 | mut/mut | 94 / 112 | 202 / 202 | 358 / 360 | 249 / 249 | 405 / 405 | 356 / 356 | 271 / 271 | 160 / 165 |
| 28 | 1975 | mut/mut | 95 / 112 | 202 / 210 | 358 / 360 | 249 / 249 | 405 / 405 | 355 / 355 | 271 / 271 | 162 / 162 |
| 29 | 1979 | mut/mut | 94 / 112 | 202 / 202 | 358 / 360 | 249 / 249 | 405 / 413 | 355 / 355 | 271 / 279 | 165 / 165 |
| 30 | 1976 | mut/wt | 95 / 110 | 202 / 210 | 358 / 360 | 249 / 249 | 405 / 405 | 355 / 365 | 271 / 271 | 164 / 164 |
| 31 | 1968 | mut/wt | 95 / 108 | 203 / 216 | 358 / 360 | 249 / 255 | 405 / 451 | 356 / 356 | 281 / 281 | 162 / 162 |
| 32 | 2000 | mut/mut | 95 / 112 | 210 / 210 | 358 / 360 | 249 / 249 | 405 / 405 | 355 / 355 | 271 / 271 | 164 / 164 |
| 33 | 2010 | mut/mut | 94 / 112 | 203 / 210 | 358 / 360 | 249 / 249 | 405 / 405 | 355 / 355 | 271 / 271 | 164 / 164 |
| 34 | 1962 | mut/mut | 94 / 115 | 202 / 202 | 358 / 360 | 249 / 249 | 422 / 422 | 354 / 354 | 271 / 281 | 162 / 162 |
| 35 | 1955 | mut/wt | 112 / 116 | 202 / 216 | 358 / 358 | 250 / 252 | 422 / 447 | 353 / 367 | 270 / 281 | 162 / 162 |
| 36 | 2001 | mut/mut | 94 / 108 | 202 / 202 | 357 / 359 | 249 / 249 | 404 / 404 | 355 / 355 | 271 / 271 | 164 / 164 |

Additionally, n=70 family members were also investigated, data not shown here, it is available upon request.

ATBp3, Antithrombin Budapest 3 mutation
